# An Automated Tracking Approach for Extraction of Retinal Vasculature in Fundus Images

**Published:** 2010-01

**Authors:** Alireza Osareh, Bita Shadgar

**Affiliations:** Computer Science Department, Shahid Chamran University, Ahvaz, Iran

**Keywords:** Blood Vessel Extraction, Pixel Classification, Neural Networks

## Abstract

**Purpose:**

To present a novel automated method for tracking and detection of retinal blood vessels in fundus images.

**Methods:**

For every pixel in retinal images, a feature vector was computed utilizing multiscale analysis based on Gabor filters. To classify the pixels based on their extracted features as vascular or non-vascular, various classifiers including Quadratic Gaussian (QG), K-Nearest Neighbors (KNN), and Neural Networks (NN) were investigated. The accuracy of classifiers was evaluated using Receiver Operating Characteristic (ROC) curve analysis in addition to sensitivity and specificity measurements. We opted for an NN model due to its superior performance in classification of retinal pixels as vascular and non-vascular.

**Results:**

The proposed method achieved an overall accuracy of 96.9%, sensitivity of 96.8%, and specificity of 97.3% for identification of retinal blood vessels using a dataset of 40 images. The area under the ROC curve reached a value of 0.967.

**Conclusion:**

Automated tracking and identification of retinal blood vessels based on Gabor filters and neural network classifiers seems highly successful. Through a comprehensive optimization process of operational parameters, our proposed scheme does not require any user intervention and has consistent performance for both normal and abnormal images.

## INTRODUCTION

Diabetes mellitus and its associated complications, including diabetic retinopathy have been identified as a growing global public health problem.^1^ About 22% to 36% of diabetic subjects suffer from retinopathy, of which one-third are considered sight-threatening.^2^ In the proliferative stage, new blood vessels are formed which emerge from the area of the optic disc spreading toward the macula or arise from peripheral vessels. Early treatment and constant monitoring of proliferative diabetic retinopathy can prevent 90% of visual loss.^3^

Monitoring includes regular retinal examinations. Digital images are obtained from the retina and graded by trained professionals. Progression of diabetic retinopathy is assessed by its severity, which in turn determines the frequency of examinations. However, a significant shortage of professional observers has prompted computer assisted monitoring. Assessment of vasculature plays an important role in a variety of medical disorders. For this purpose, a number of parameters need to be measured which include vascular caliber, color, reflectivity, tortuosity and abnormal branching. When the number of vessels in any single image is large, or when a large number of images is acquired, manual delineation of vessels becomes tedious or even impossible.

Manifestations of several vascular disorders, such as diabetic retinopathy, depend on analysis of the vascular network. Retinal vessel segmentation can simplify screening for retinopathy by reducing the number of false positive results in microaneurysm detection and may serve as a means of image registration from the same patient taken at different times by delineating the location of the optic disc and fovea. However, manual detection of blood vessels is not simple because the vessels in a retinal image are complex and have low contrast; this underscores the need for a reliable automated method for extracting and measuring vessels in retinal images.

The current study is part of a larger effort to develop an automated screening system for identification of diabetic retinopathy.^4^–^6^ Non-mydriatic camera images were analyzed in an attempt to fine tune and improve the quality of blood vessel tracking. Here, we used the wavelet as a tool for segmentation of retinal blood vessels in combination with feature extraction and classification of retinal image pixels. The advantage of wavelet analysis is its multiscale analyzing capability in tuning to specific frequencies, allowing noise filtering and blood vessel enhancement in a single step. We also describe the properties of our images and discuss our proposed method.

## METHODS

Our proposed approach was implemented on the DRIVE dataset.^7^ This database contains 40-color retinal images and has been divided into a training and a test set, each containing 20 images. For the training images, single manual segmentation of the vasculature is available. For the test cases, two manual segmentations are available; one is used as a gold standard and the other is used to compare computer generated segmentations with those by an independent human observer. All human observers that manually segmented the vasculature were instructed and trained by an experienced ophthalmologist. They were asked to mark all pixels which they were sure for 70% or more to be vessels.

**Figure 1:**
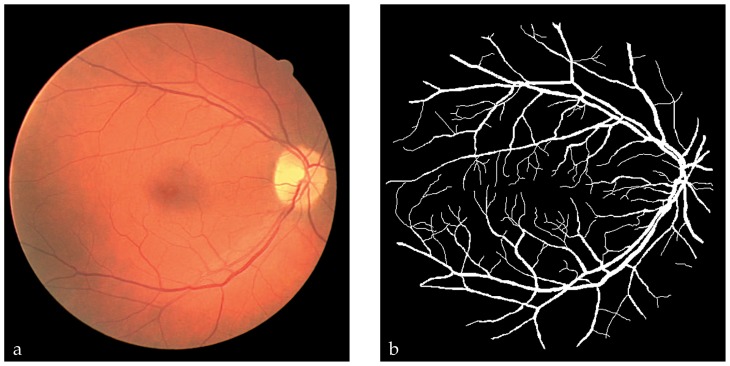
Manual vessel segmentation: **(a)** a typical retinal image, **(b)** manually segmented vessels.

Some images were obtained from patients with no ocular pathology (normal subjects); others contained pathologies such as microaneurysms, hemorrhages and exudates which can obscure or confuse blood vessel appearance in varying parts of the image. All images were obtained by a Canon CR5 non-mydriatic retinal camera (Canon, Kanagawa, Japan) with a 45° field of view. Images were 565×584 pixels in size at 24-bit red, green and blue (RGB). Figure 1(a) shows a typical retinal image, its corresponding manual vessel segmentation is displayed in Figure 1(b).

The purpose of this study was to classify every retinal image pixel automatically as vascular or non-vascular. For this purpose, we needed labeled pixels or training sets, features and classifiers. We assumed a binary multi-dimensional classification approach to distinguish vascular pixels from non-vascular ones including other anatomico-pathological structures and artifacts. Our chosen data for the learning stage consisted of typical pixels, which were representative of our classification problem. To make up such a dataset, examples including vessels and non-vessels were labeled manually and then used to train and test the classifiers (see Figure 1). The obtained labeled examples (feature vectors) were then mapped into the feature space, and their labels were utilized to obtain subspaces corresponding to the two different classes, i.e. vascular and non-vascular.

Gabor filters are powerful tools that have been widely used for multi-scale/multi-directional analysis in image processing. These filters have shown high performance as feature extractors for discrimination purposes. Due to the directional selectiveness of Gabor filters in detecting oriented features and fine tuning to specific orientations and scales, these filters act as low-level oriented edge discriminators. Methods based on these filters have been very successful in iris recognition,^8^ face recognition,^9^ and have provided state-of-the-art accuracy in fingerprint matching.^10^ By selectively changing Gabor parameters, namely orientations and scales, we were able to tune the filter response to particular patterns such as blood vessels.

Having primarily extracted the candidate blood vessel network based on Gabor filter responses, image pixels were classified as vascular and non-vascular. For this purpose, we had to represent these pixels using relevant features that produced best-class separability. The feature set should be selected such that inter-class discrimination is maximized while intra-class discrimination is minimized. In order to avoid facing a high dimensional feature space it is desirable to work with a more compact feature set.

The pixel feature space was constituted by maximum Gabor filter responses of all orientations taken at different scale values. To take other important retinal image features such as color and local information toward a more effective differentiation of pixels, a square window was centered on each underlying pixel in the image. Then the L*uv* color components of all pixels in the window were directly composed into the feature vector of the pixel of interest. We experimented with a number of color spaces including RGB, HIS, Lab, and L*uv* and found that color spaces which separate luminance and chrominance (for example, L*uv*) are more suitable for this purpose.^11^ There might be no constraint on the window size in theory, but it is assumed that most local information is presented in a small neighborhood of the pixel of interest. Thus the window must be chosen large enough to contain blood vessels, but also small enough to avoid interference from neighboring non-vessel pixels.

To classify the image pixels in terms of their extracted features as vascular or non-vascular, various classifiers including Quadratic Gaussian (QG), K-Nearest Neighbors (KNN), and Neural Networks (NN) were investigated.^12^ It should be noted that, manually segmented images such as Figure 1(b) were used both to construct learning datasets for vascular and non-vascular pixels and to train the above-mentioned classifiers.

To initially extract the vessels from retinal images (coarse stage), we used a set of Gabor filters arranged in 18 orientations (θ spanning from 0° up to 170° in 10° steps) and 4 scales (σ=3, 5, 7, 9). The scale values were experimentally tuned according to our prior knowledge of retinal image characteristics and to assign stronger responses to pixels associated with blood vessels. For each considered set of scale parameters, we were interested in Gabor filter response with maximum values over all possible orientations. These values were then taken as the main components of the pixel feature vectors.

To determine the optimal window size for extracting the most relevant local information of the image, we examined various sizes and obtained the best results with a 3×3 window size. Here, a balanced dataset of vascular and non-vascular pixels was established to eliminate any possible bias towards either of the two classes. Our representative learning dataset comprised of 125,000 vessel and 125,400 non-vessel pixels randomly collected from 20 retinal images. This dataset was then divided into a training set, a validation set, and a test set in a 65:10:25 ratio.

We employed a three-layer perceptron NN with a 15 node input layer corresponding to our feature vector. We experimented with a hidden layer with a range of 2 to 20 hidden units to find the optimum architecture. A single output node gave the final classification probability. The network was trained using standard Back-Propagation learning method.^12^ The classifier error was calculated using pixels from validation set after each iteration of training. The NN performance was measured using the previously unseen features from the test set in the usual terms of sensitivity and specificity. Sensitivity is the ratio of true positive (TP) decisions to all positive decisions, while specificity is the ratio of true negative (TN) decisions to all negative decisions.^12^ Another reported measure, the accuracy, is the ratio between the total number of correctly classified vascular pixels to all instances existing in the test set.

**Figure 2:**
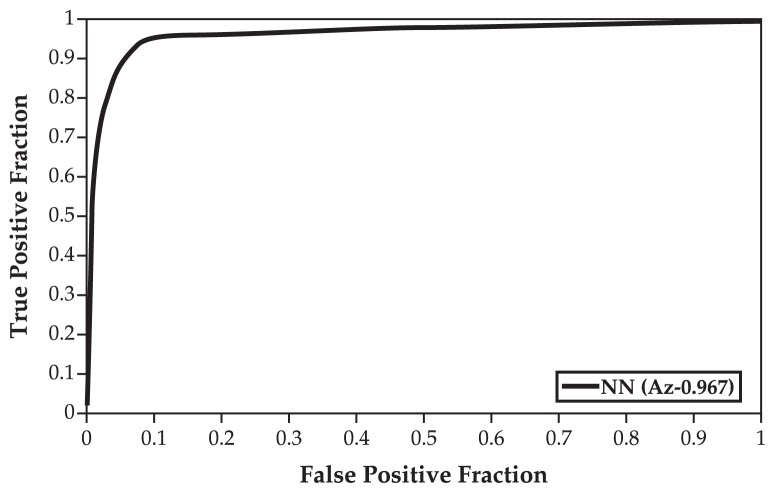
Optimum neural network (NN) based receiver-operating characteristic curve.

The classical tool to achieve tradeoff between sensitivity and specificity criteria is the Receiver Operating Characteristics (ROC) curve.^13^ This curve is typically plotted with the TP fraction against the FP fraction. The bigger the area under the ROC curve (*A_z_*), the higher the probability of making a correct decision. Figure 2 compares the behavior of the optimum NN classifier for the full range of output threshold values. The optimum NN classifier achieved very good performance with *A_z_* value of 0.967. Thus, we opted for this optimum NN model due to its superior performance in classification of retinal pixels into vascular and non-vascular classes.

## RESULTS

**Figure 3:**
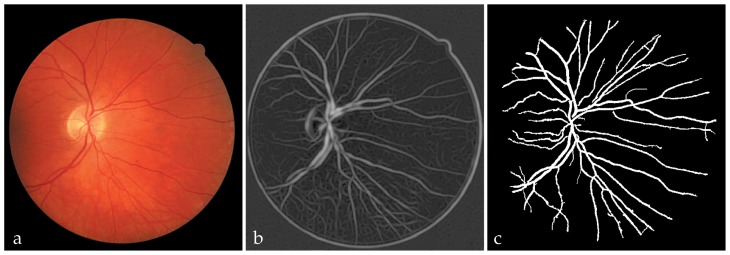
Maximum Gabor filter response for different orientations: **(a)** a typical retinal image, **(b)** maximum Gabor filter response (σ =7), **(c)** neural network based classified vessels.

Figure 3(b) shows the corresponding maximum Gabor filter response for the image shown in Figure 3(a) where σ was chosen to be 7.

Table 1 compares classification performances for two other classifiers, i.e. KNN and QG against the best results obtained with the NN. The combination of selected features provided a good classification performance for all the classifiers. Overall, the classification analysis indicated that the best optimum classifier for distinguishing vascular pixels is a NN classifier with 10 hidden units. The second best performance was achieved by KNN classifier, while QG yielded the lowest performance.

**Figure 4:**
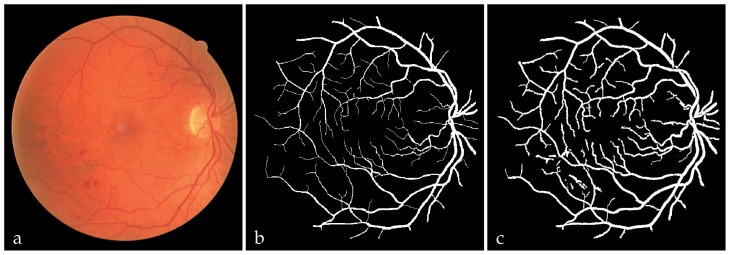
Extracted blood vessels: **(a)** a typically abnormal retinal image, **(b)** manually labeled vessels, **(c)** neural network based classified vessels.

Figure 3(c) illustrates the final segmented vessels identified based on the proposed approach. Similarly, Figure 4 illustrates a typical abnormal retinal image from the image dataset that has been classified at pixel level using the optimum NN classifier. The manually segmented vessels and the final extracted blood vessels are also shown in this figure. Although, the majority of large and small vessels were detected, there was erroneous detection of noise and other artifacts. The majority of errors were due to background noise and non-uniform illumination across the retinal images, the border of the optic disc and other types of pathology such as false positive pixels in Figure 4(c) that present strong contrast. Another difficulty was the lack of precision in capturing some of the thinnest vessels that are barely perceived by human observers. In fact, small retinal vessels usually have poor local contrast and almost never have ideal solid edges.

## DISCUSSION

Retinal blood vessel segmentation is the key step in diabetic retinopathy screening systems because vessels act as the landmarks for other structures such as the optic disc and fovea. This requires reliable automated detection of blood vessels preserving various vessel measurements. In the past years, several approaches for extracting retinal image vessels have been developed which can be divided into two groups; one consists of supervised classifier-based algorithms and the other utilizes tracking-based approaches. Supervised classifier-based algorithms usually comprise of two steps. First, a low-level algorithm produces segmentation of spatially connected regions. These candidate regions are then classified as vascular or non-vascular. In a study by Tamura et al^14^, regions segmented by a user-defined threshold were classified as vascular or lesion according to their length-to-width ratio. In the study by Leandro et al^15^ the application of mathematical morphology and wavelet transform was investigated for identification of retinal blood vessels. In a follow-up study, a two-dimensional Gabor wavelet was utilized to initially segment the retinal images. A Bayesian classifier was then applied to classify extracted feature vectors as vascular or non-vascular.^16^

Tracking-based approaches utilize a profile model to incrementally step along and segment a vessel. In another study by Tamura et al^17^, a Hough transform was used to locate the papilla in retinal images. Vessel tracking proceeded iteratively from the papilla, halting when the response to a one-dimensional matched filter fell below a given threshold. In the study by Tolias and Panas^18^, the tracking method was driven by a fuzzy model of a one-dimensional vessel profile. One drawback to these approaches is their dependence upon unsophisticated methods for locating the starting points, which must always be either at the optic nerve or at subsequently detected branch points. In a paper by Zana and Kelin^19^, blood vessels were detected by means of mathematical morphology. In the study by Al-Rawi and Karajeh^20^, matched filters were applied in conjunction with other techniques such as genetic algorithms and piecewise thresholding.

In the current study, we presented a novel automated blood vessel extraction technique using Gabor filter-based extracted features and NN classifiers. Our results suggest that pixel-level classification in conjunction with Gabor filter responses, feature extraction and NN classifiers can provide robust blood vessel segmentation while suppressing the background. Apart from NN classifiers, we investigated other classifiers such as KNN and QG. We found that an NN based on the back-propagation learning method could provide the best overall diagnostics with 96.9% accuracy, 96.8% sensitivity, and 97.3% specificity, where the trade-off between sensitivity and specificity was appropriately balanced for this particular application.

In order to compare our results with the most relevant works in the literature, the performance of five different algorithms all of which had been evaluated using DRIVE dataset were compared.^16^,^21^,^22^ Table 2 shows an overview of the results for different methods in terms of the area under the ROC curve (*A_z_*). As evident, the area under the ROC curve in our method reached a value of 0.967 and is favorably comparable and to some extent higher than previously reported accuracies which range from 0.787 to 0.961.

In conclusion, our proposed vessel extraction technique does not require any user intervention, and has consistent performance in both normal and abnormal images. A perfect medical system would yield an area under ROC curve of 1, however, a value of 0.967 in our study is higher than that of other previously reported vessel segmentation methods. The results demonstrated herein indicate that automated identification of retinal blood vessels based on Gabor filter responses and NN classifiers can be very successful. Hence, eye care specialists can potentially monitor larger populations using this method. Furthermore, observations based on such a tool would be systematically reproducible.

## Figures and Tables

**Table 1:** Optimum classification results for different classifiers (values in %)

Classifier	Specificity	Overall Accuracy	Sensitivity
KNN (K=5)	91.9	92.6	93.5
QG	88.3	89.1	90.0
NN	97.3	96.9	96.8

KNN, K-Nearest Neighbors; QG, Quadratic Gaussian; NN, Neural Networks

**Table 2:** Comparison of different blood vessel identification techniques in terms of A_z_

Blood Vessel Extraction Method	Area under the ROC Curve (A_z_)
Chaudhuri et al^21^	0.787
Zana & Kelin^23^	0.898
Jiang & Mojon^22^	0.911
Niemeijer et al^24^	0.929
Soares et al^16^	0.961
Current study	0.967

ROC, receiver-operator characteristic.
